# The Effect of Dexmedetomidine on the Mini-Cog Score and High-Mobility Group Box 1 Levels in Elderly Patients with Postoperative Neurocognitive Disorders Undergoing Orthopedic Surgery

**DOI:** 10.3390/jcm12206610

**Published:** 2023-10-19

**Authors:** Seung Hee Yoo, Mi Jin Jue, Yu-Hee Kim, Sooyoung Cho, Won-joong Kim, Kye-Min Kim, Jong In Han, Heeseung Lee

**Affiliations:** 1 Department of Anesthesiology and Pain Medicine, College of Medicine, Ewha Womans University, Ewha Womans University Mokdong Hospital, Seoul 07985, Republic of Korea; shyoo0710@ewha.ac.kr (S.H.Y.); iljmj52@hanmail.net (M.J.J.); sooyoung.cho@ewha.ac.kr (S.C.); ickypoo@ewha.ac.kr (W.-j.K.); hanji@ewha.ac.kr (J.I.H.); 2 Advanced Biomedical Research Institute, Ewha Womans University Seoul Hospital, Seoul 07804, Republic of Korea; kimyuhee@ewha.ac.kr; 3 Department of Anesthesiology and Pain Medicine, Inje University Sanggye Paik Hospital, Seoul 01757, Republic of Korea; kyemin@paik.ac.kr

**Keywords:** aged, dexmedetomidine, HMGB1, Mini-Cog, neurocognitive disorders, orthopedic surgery, postoperative cognitive complications

## Abstract

Dexmedetomidine prevents postoperative cognitive dysfunction by inhibiting high-mobility group box 1 (HMGB1), which acts as an inflammatory marker. This study investigated the HMGB1 levels and the cognitive function using a Mini-Cog© score in elderly patients undergoing orthopedic surgery with dexmedetomidine infusion. In total, 128 patients aged ≥ 65 years were analyzed. The patients received saline in the control group and dexmedetomidine in the dexmedetomidine group until the end of surgery. Blood sampling and the Mini-Cog© test were performed before the surgery and on postoperative days 1 and 3. The primary outcomes were the effect of dexmedetomidine on the HMGB1 levels and the Mini-Cog© score in terms of postoperative cognitive function. The Mini-Cog© score over time differed significantly between the groups (*p* = 0.008), with an increase in the dexmedetomidine group. The postoperative HMGB1 levels increased over time in both groups; however, there was no significant difference between the groups (*p* = 0.969). The probability of perioperative neurocognitive disorders decreased by 0.48 times as the Mini-Cog© score on postoperative day 3 increased by 1 point. Intraoperative dexmedetomidine has shown an increase in the postoperative Mini-Cog© score. Thus, the Mini-Cog© score is a potential tool for evaluating cognitive function in elderly patients.

## 1. Introduction

The number of geriatric patients undergoing surgery under general anesthesia has increased steadily with the advent of an aging society, resulting in growing medical expenses [1]. Perioperative neurocognitive disorders (PND) encompass overall cognitive decline, including postoperative delirium (POD), delayed neurocognitive recovery, and postoperative cognitive dysfunction (POCD), that occurs between the pre- and postoperative periods [2]. POD, which can occur in up to 50% of patients undergoing surgeries and is associated with increased morbidity and mortality, can result in long-term outcomes, such as functional and cognitive decline [2,3,4,5,6]. Gou et al. reported that POD leads to approximately USD 32.9 billion of additional healthcare costs in the United States per year, constituting a remarkable international health issue that cannot be ignored [7]. In addition, POD can lead to POCD without prompt management [4]. Therefore, the identification of patients at risk of developing POD and the prevention of POCD has received considerable attention in recent years as it is crucial for the early recognition and strategic management of these patients by clinicians and caregivers [8]. 

Several studies have demonstrated a relative risk reduction of 30–40% for cognitive declines in non-pharmacological and pharmacological trials [9,10,11,12]. It has also been reported that POCD occurs due to changes in the systemic inflammatory response caused by stressful situations, such as pain and the administration of drugs during surgery and for the induction and maintenance of anesthesia [5,13,14], as well as non-modifiable factors, such as age [5,6,13], sex [4], dementia or cognitive dysfunction [5,6,13], and underlying comorbidities [5,6,15]. Nevertheless, reliable and robust mechanisms for the development and prevention of cognitive impairment have not been established [16]. Establishing a convenient clinical tool for diagnosing perioperative cognitive function is a requirement for anesthesiologists working in urgent settings. The Mini-Cog© test, which requires 2–3 min to complete, is a neuropsychological test that is used as a fast screening tool to detect cognitive decline [17,18]. It has also been used to predict cognitive function in elderly patients undergoing surgery [17,19,20].

The high-mobility group box 1 (HMGB1) protein, a proinflammatory cytokine that is released during an immune response, is capable of increasing the permeability of the blood–brain barrier (BBB) [21,22,23]. Numerous studies have implicated the role of HMGB1-induced inflammatory response in cognitive decline following aseptic surgical trauma, and preclinical studies have demonstrated the alleviation of HMGB1-induced neuroinflammation via the administration of anti-HMGB1 antibodies [24,25]. Thus, studies have highlighted its biological function through its pivotal role as an inflammatory marker [21,26].

Intraoperative dexmedetomidine infusion reduces the severity of POD by mitigating surgery-related neuroinflammatory responses [27,28,29]. Animal experiments have also revealed the neuroprotective effect of dexmedetomidine through the inhibition of HMGB1 [30,31].

To the best of our knowledge, no randomized controlled trials in humans have evaluated the effect of the administration of dexmedetomidine on HMGB1 levels in relation to cognitive decline. Therefore, we hypothesized that the infusion of dexmedetomidine can reduce the incidence of PND and aimed to investigate the HMGB1 levels and evaluate the perioperative cognitive function using the Mini-Cog© score in elderly patients undergoing orthopedic surgery under general anesthesia with or without dexmedetomidine infusion.

## 2. Materials and Methods

This double-blind, randomized, prospective trial was conducted at the Ewha Womans University Medical Center, Seoul, Korea, between September 2021 and September 2022. This study was approved by the Institutional Review Board (EUMC 2021-05-036-011, approval on 4 August 2021) and registered with the Clinical Trial Registry of Korea (KCT 0006497, posted on 6 August 2021). We also conducted this study in accordance with the Declaration of Helsinki, 2013. All patients provided written informed consent before the commencement of this study.

### 2.1. Patients and Randomization

The inclusion criteria were patients aged ≥ 65 years scheduled to undergo elective orthopedic surgery lasting > 2 h under general anesthesia, those requiring more than three days of hospital stay after the surgery, and those capable of reading or writing. The exclusion criteria were as follows: previously diagnosed dementia, features of delirium, allergy to dexmedetomidine, hearing or speaking difficulties, illiteracy, alcohol-related disorders, American Society of Anesthesiologists physical status (ASA-PS) IV or V, admission to the intensive care unit, and refusal to provide informed consent at any time during the study period.

The patients were divided into Group C (control group) or Group D (dexmedetomidine group) according to the randomization allocation program using a block size of eight. The randomization envelopes marked with the group and sequential numbers were disclosed along with the patient number on the day of the surgery by another anesthesiologist. The study drug was prepared in a 50 mL syringe by an anesthetic nurse according to the randomization sequence. To fulfill the implementation of the “double-blind”, we conducted a study in a manner where there was blinding between the anesthesiologist and the medical staff responsible for blood sampling, as well as between the anesthesiologist and the medical staff administrating the mini-Cog test.

### 2.2. Study Protocol

**Figure 1:**
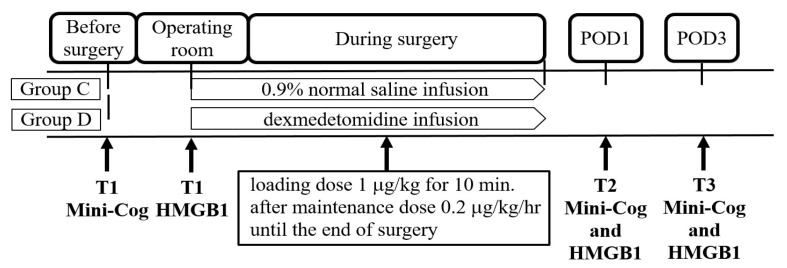
Schematic diagram of the study. POD 1, postoperative day 1; POD 3, postoperative day 3; T1, before the surgery; T2, postoperative day 1; T3, postoperative day 3.

The patients allocated to Group D received an infusion of dexmedetomidine (50 mL for 200 μg at a dose of 4 μg/mL of dexmedetomidine hydrochloride in 0.9% sodium chloride injection; Pfizer Pharmaceuticals Korea Ltd., Seoul, Republic of Korea), whereas those allocated to Group C received an infusion of 0.9% normal saline. The study drug was administered using a syringe pump (Terufusion™ Syringe Pump Smart, Therumo TE-SS700, Terumo Co., Ltd., Tokyo, Japan) that could infuse the dosage precisely (Figure 1).

Anesthesia monitoring, including noninvasive blood pressure, pulse oximetry, and electrocardiography, was initiated when the patients arrived at the operating room. Blood sampling was performed immediately after monitoring. A loading dose of 1 μg/kg was administered for 10 min after blood sampling, and a maintenance dose was administered at a rate of 0.2 μg/kg/h until the end of the surgery. 

All patients received 1–2 mg/kg of 1% propofol, 0.5–1 μg/kg of fentanyl, and 0.6 mg/kg of rocuronium to facilitate endotracheal intubation for the induction of anesthesia. Propofol was titrated according to the response received to calling the patient’s name until the patient lost consciousness. Anesthesia was maintained by administering sevoflurane at a concentration of 1–2.5% with a fraction of inspired oxygen of 50% in fresh gas at 3 L/min via a circle anesthesia breathing system. Fentanyl was administered for intraoperative additive analgesia when the heart rate increased to >20 beats/min, and rocuronium was administered for muscle relaxation. During the operative period, the patients were ventilated with a tidal volume of 6–8 mL/kg for ideal body weight, positive end-expiratory pressure of 5 cm H_2_O, and respiratory rate of 10–14 times/min and maintained with the goal of an end-tidal carbon dioxide pressure of 35–40 mmHg. The depth of anesthesia was maintained at a bispectral index of 40–60. The infusion of the study drugs was discontinued at the end of the surgery. If the train-of-four count was 2–4, 200 mg of sugammadex was administered to counteract the effect of rocuronium, after which the patients were extubated. A bolus of 5 mg of ephedrine was administered perioperatively if the mean blood pressure was <65 mmHg or if the blood pressure was reduced by >20% of the baseline. Postoperative pain was controlled using intravenous patient-controlled analgesia (IV-PCA) and a PCA pump (AutoMed^®^ 3200 module, ACE Medical. Co., Seoul, Republic of Korea). A PCA device, consisting of 100 mL of PCA comprising 500 μg of fentanyl and 0.3 mg of ramosetron in 48 mL of normal saline, was connected to the patient. It was delivered at a background flow rate of 0.5 mL/h, with a demand bolus of 0.5 mL and a lockout period of 15 min.

### 2.3. Measurement of HMGB1

Venous blood samples were obtained from all participants at three different time points: preoperative (T1), blood samples were collected from patients in both groups upon entering the operating room before anesthetic induction; postoperative day 1 (T2); and postoperative day 3 (T3) (Figure 1). Blood samples were obtained from the venous blood in the serum separation tubes immediately after blood collection. The tubes were gently inverted 8–10 times and centrifuged for 15 min at 4000 rpm at 25 °C. After centrifugation, the serum of the supernatants was transferred to aliquots of 1.5 mL and immediately stored at −80 °C for analysis. The HMGB1 levels were measured using an enzyme-linked immunosorbent assay (HMGB1 Express ELISA kit; REF. 30164033; IBL, Hamburg, Germany) according to the manufacturer’s instructions.

The brief steps of the HMGB1 assay were as follows: Samples stored at −80 °C were thawed. After pipetting the standard (standard 1–7), positive control, and samples into each well of the microtiter plate, the plate was covered with adhesive foil and incubated for 2 h at 37 °C to bind the HMGB1 antibody on the wells. After washing the plate, 100 μL of the enzyme conjugate was pipetted into each well using an eight-channel micropipette and incubated for 1 h at 25 °C. Subsequently, the plate was washed five times, and 100 μL of the color solution was pipetted into each well and incubated for 20 min at 25 °C. Lastly, 100 μL of the stop solution was added to the well to terminate the color reaction. The optical density was measured at a wavelength of 450 nm, and the concentration values obtained were calculated using a fitted standard curve.

### 2.4. Mini-Cog© Test

The incidence of POCD was determined using the Mini-Cog© test (Supplementary S1) at three time points: T1, T2, and T3. A trained physician visited and assessed the neurological function of the patients using the Mini-Cog© test (Figure 1). The scores of the three-item recall test were presented as a range of 0–3, whereas those of the clock drawing test (CDT) were presented as a range of 0–2. The scoring system was combined with the sum of both tests, and the total score was 5. A cut-off score of <4 in the Mini-Cog© test was used to assess cognitive function. A score of 0–3 indicated positive cognitive decline screening, whereas a score of 4–5 indicated negative cognitive decline screening.

### 2.5. Outcome Measurement

The serum HMGB1 level and Mini-Cog© test score were measured at three time points, T1, T2, and T3, for each patient.

The collected data included patient demographics (age; sex; height; weight; body mass index [BMI]; ASA-PS; underlying diseases, such as diabetes mellitus, hypertension, chronic obstructive pulmonary disease, and renal disease; and educational level). The intraoperative variables included the total amount of infused study drugs and propofol, total dose of fentanyl, use of vasopressors, total duration of surgery and anesthesia, total input, and estimated blood loss. The postoperative data included the prevalence of delirium according to CAM-ICU (Supplementary S2) on postoperative days 1 and 3, the numeric rating scale (NRS) pain score on postoperative day 1, postoperative nausea and vomiting (PONV), and the total fentanyl dose of PCA. 

The primary outcomes assessed in this study were the effect of dexmedetomidine on the serum HMGB1 levels and the Mini-Cog© score in terms of PND before the surgery and on postoperative days 1 and 3 compared with that in the control group. The secondary outcomes included the comparison of postoperative pain scores and the total doses of anesthetics (propofol) and analgesics administered, including IV-PCA drugs (fentanyl).

### 2.6. Statistical Analysis

The sample size was determined based on previous studies on the serum HMGB1 levels [32], assuming that there was a mean difference of 8 ng/mL in the HMGB1 levels at T3 between the two groups, with a standard deviation of 14 ng/mL, an alpha value of 0.05, and a power of 0.8. The required sample size to detect differences between the groups was 128 patients, and the analysis was performed using the G power software 3.1 version. 

Data are presented as means ± standard deviation (SD) for continuous variables and as counts or % for categorical variables. Numerical variables and HMGB1 levels between the groups were analyzed using the Student’s *t*-test for normally distributed data, and ordinal variables were analyzed using the Mann–Whitney U-test. Categorical variables and the incidence of delirium were analyzed using Fisher’s exact and chi-square tests. All variables were analyzed using the Kolmogorov–Smirnov test for normality.

The repeated measures of two-way analysis of variance (ANOVA) followed by the Bonferroni test for post hoc comparison was performed to compare the differences in the HMGB1 levels and Mini-Cog© score over time between groups C and D. Multivariate logistic regression analysis was performed after univariate logistic regression analysis to identify the variables associated with PND. The variables included all covariates with *p* < 0.05 from the univariate analysis, such as ASA-PS, underlying diabetes mellitus, T2 Mini-Cog© score, T3 Mini-Cog© score, and T3 HMGB1 levels. Furthermore, the educational level and duration of anesthesia, depending on the administration of dexmedetomidine, were included. The parameters were tested for multicollinearity using the variance inflation factor (VIF) and a correlation matrix, and the results are expressed as odds ratios (OR) and 95% confidence intervals (CIs). Statistical analysis was performed using Statistical Package for the Social Sciences (SPSS) version 25 (IBM Corp., Armonk, NY, USA). Statistical significance was set at *p*-value < 0.05.

## 3. Results

**Figure 2:**
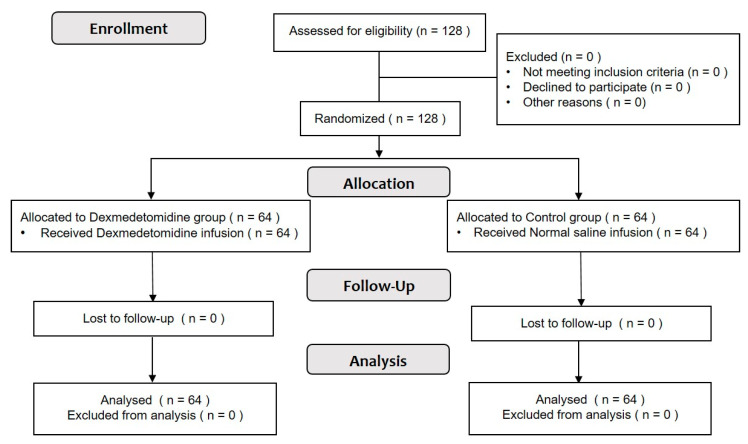
The CONSORT flow diagram.

In total, 128 patients who underwent orthopedic surgery under general anesthesia were included in this study and randomly allocated to two groups: Group C (*n* = 64) and Group D (*n* = 64). A flow diagram of the Consolidated Standards of Reporting Trials (CONSORT) is presented in (Figure 2).

### 3.1. Baseline Patient Characteristics

The demographic characteristics, including age, sex, BMI, ASA-PS, educational level, length of hospital stay, and underlying disease, showed no significant differences between the groups (Table 1).

### 3.2. Perioperative Variables

Table 2 presents the perioperative variables. The total doses of the study drugs, the total doses of fentanyl and propofol, the number of patients who received vasopressors, the total duration of surgery and anesthesia, the total volume of fluid administered, and the estimated blood loss were intraoperative variables. The intraoperative variables were fairly matched between the two groups, except for the total doses of propofol and fentanyl and the number of patients who received vasopressors. The number of patients who received intraoperative vasopressors was higher in Group D (68.75%, 44/64) than in Group C (42.19%, 27/64) (*p* = 0.002). PND and PONV were the postoperative variables. PND occurred in 14 patients, accounting for 15.63% (10/64) in Group C and 6.25% (4/64) in Group D (*p* = 0.089), indicating that the difference was not significant between the groups. Furthermore, there was no significant difference in the incidence of PONV between the groups (*p* = 0.849).

### 3.3. Primary Outcomes

**Figure 3:**
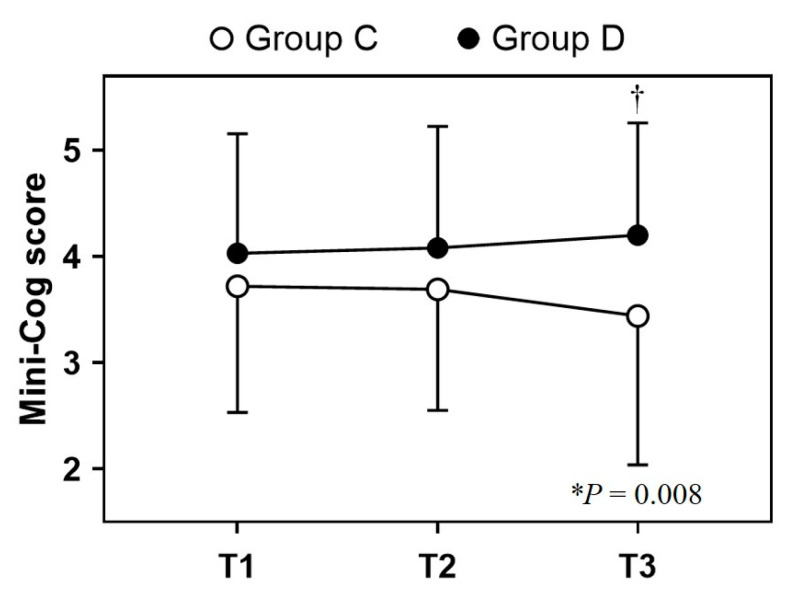
Analysis of the Mini-Cog© scores. The values are presented as mean ± SD. Repeated measures of two-way analysis of variance (ANOVA) and Bonferroni post hoc test were performed for the Mini-Cog© score. When comparing the groups, the Mini-Cog© score differed significantly over time (*p* = 0.008). The difference in the Mini-Cog© score in Group D increased over time, whereas that in Group C decreased over time. The Mini-Cog© scores at T3 were significantly higher in Group D than those in Group C; however, the scores at T1 and T2 were comparable between the groups (T1, *p* = 0.129; T2, *p* = 0.055; T3, *p* = 0.001). T1, before the surgery; T2, postoperative day 1; T3, postoperative day 3; Group C, control group; Group D, dexmedetomidine group; * *p* = 0.008 (intergroup difference over time). † *p* = 0.001 (inter-group difference from T2 to T3).

A comparison of inter-group differences revealed that the changes in the Mini-Cog© score over the study period showed a statistically significant difference between the groups, indicating that the Mini-Cog© score in Group D showed an increasing pattern, whereas that in Group C showed a decreasing pattern over time despite both groups having similar preoperative Mini-Cog© scores (*p* = 0.008). Following the Bonferroni test for post hoc analysis, the T3 Mini-Cog© score of Group D differed significantly from that of Group C (*p* = 0.001) (Figure 3). The Mini-Cog© scores differed significantly between the two groups at each time point: 3.72 ± 1.19 vs. 4.03 ± 1.13, *p* = 0.129 at T1; 3.69 ± 1.14 vs. 4.08 ± 1.15, *p* = 0.055 at T2; 3.44 ± 1.40 vs. 4.20 ± 1.06, *p* = 0.001 at T3 in Group C and Group D, respectively. Comparison of the intra-group differences revealed that the differences in the Mini-Cog© scores were comparable between two time points: 0.031 vs. 0.047 from T1 to T2, 0.281 vs. 0.172 from T1 to T3, and 0.250 vs. 0.125 from T2 to T3 in Group C and Group D, respectively (Table 3, upper panel).

**Figure 4:**
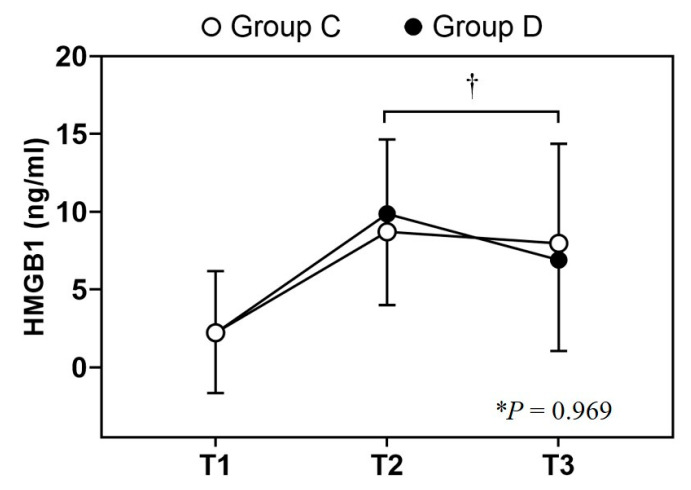
Analysis of the serum HMGB1 levels. The values are presented as mean ± SD. Repeated measures of two-way analysis of variance (ANOVA) and Bonferroni post hoc test were performed for the serum HMGB1 levels. When comparing the groups, the changes in the serum HMGB1 levels did not differ significantly over time (*p* = 0.969). The HMGB1 levels at T2 and T3 were significantly higher than those at T1 in both groups. The mean difference in the HMGB1 levels between T2 and T3 was statistically significant in Group D (*p* = 0.004) but not in Group C (*p* = 1.000). T1, before the surgery; T2, postoperative day 1; T3, postoperative day 3; Group C, control group; Group D, dexmedetomidine group; * *p* = 0.969 (intergroup difference over time). † *p* = 0.004 (comparison from T2 to T3 in Group D).

A comparison of inter-group differences revealed that the overall change in the HMGB1 levels was similar in both groups (*p* = 0.969). Following the Bonferroni post hoc test, the differences in the HMGB1 levels over time were similar in both groups (Figure 4). The mean HMGB1 levels at each time point in both groups were similar: 2.22 ± 3.96 ng/mL vs. 2.21 ± 3.87 ng/mL at T1, 8.71 ± 5.94 ng/mL vs. 9.87 ± 5.87 ng/mL at T2, and 7.97 ± 6.40 ng/mL vs. 6.90 ± 5.86 ng/mL at T3, in Group C and Group D, respectively (Table 3, middle panel). A comparison of the intra-group differences revealed that the mean postoperative HMGB1 levels were significantly higher than the preoperative level in both groups. The HMGB1 levels on postoperative days 1 (all groups: *p* < 0.001) and 3 (all groups: *p* < 0.001) were significantly higher than the preoperative levels in both groups. The HMGB1 levels in both groups peaked on postoperative day 1 and subsequently decreased until postoperative day 3 (T1 <<< T2 > T3), indicating that the HMGB1 level on T3 was higher than the HMGB1 level on T1. Additional analysis was performed using the absolute difference of HMGB1 levels between two time points. The ∆(T1 − T2) HMGB1 levels were 6.488 ng vs. 7.658 ng/mL, the ∆(T1 − T3) HMGB1 levels were 5.745 ng/mL vs. 4.696 ng, and the ∆(T2 − T3) HMGB1 levels were 0.743 ng/mL vs. 2.963 ng in Group C and Group D, respectively. A marked decline in the ∆(T2 − T3) HMGB1 levels was observed (*p* = 0.004) in Group D compared with that in Group C (*p* = 1.000), indicating a distinct disparity between the groups (Table 3, bottom panel).

A comparison of the incidence of PND, defined as delirium in both groups using univariate analysis, revealed that ASA-PS, underlying diabetes mellitus, and the T2 and T3 Mini-Cog© scores were statistically significant. Multivariate analysis including these variables revealed that ASA-PS and the T3 Mini-Cog© score were statistically significant as independent factors (odds ratio [95% CI], 4.68 [1.29–17.01], *p* = 0.019 for ASA-PS) (odds ratio [95% CI], 0.48 [0.26–0.88], *p* = 0.017 for T3 Mini-Cog©) (Table 4). The results demonstrated that the probability of the incidence of PND would decrease by 0.48 times as the T3 Mini-Cog© score increased by 1 point.

### 3.4. Secondary Outcomes

The secondary outcomes were the comparison of postoperative day 1 pain scores and the total doses of anesthetic and analgesic drugs, including IV-PCA (Table 2). No statistically significant difference was observed between the T2 pain scores of the two groups (*p* = 0.574). The patients in Group D received significantly lower doses of propofol, fentanyl, and fentanyl administered via PCA than those in Group C. The total dose of propofol in Group D and Group C was 59.84 ± 16.67 mg and 74.38 ± 23.36 mg, respectively (*p* < 0.001). The total dose of fentanyl in Group D and Group C was 109.45 ± 32.94 μg and 141.51 ± 57.61 μg, respectively (*p* < 0.001). The total dose of fentanyl administered via PCA was 338.99 ± 104.04 μg and 277.56 ± 133.03 μg in Groups C and D, respectively (*p* = 0.005).

## 4. Discussion

This present study holds three key implications. First, the Mini-Cog© test, which has gained popularity as a screening tool for predicting cognitive impairment in elderly patients, was used in this study [32,33]. It can predict POCD without using any special tools and is less affected by educational status, age, and medical conditions [34]. The Perioperative Brain Health Initiative, an international perioperative neurotoxicity working group developed by the ASA, concurred with the statement that “Baseline cognition should be objectively evaluated with a brief screening tool preoperatively” [8]. The Mini-Cog© test is easier to perform, and no patients in this present study declined to undergo the Mini-Cog© test. However, there was a risk of memorization due to its simplicity. Therefore, we selected different versions of the word recall test and identified inter-group differences to reduce this drawback. Anesthesiologists can use this test efficiently in pre- and postoperative settings where the number of elderly patients receiving general anesthesia is increasing. Unlike other studies that performed the Mini-Cog© test only preoperatively, we performed the test at three different time points, considering the crucial factor of convenience in completing the test [19,34]. Second, this prospective randomized controlled study aimed to investigate the effect of dexmedetomidine on the cognitive function of the Mini-Cog© from a clinical perspective and HMGB1 levels from a cellular-level perspective. Third, our study is the first study to reveal the preventive effects of dexmedetomidine on HMGB1 and cognitive function throughout the perioperative period in elderly patients undergoing orthopedic surgery.

The dexmedetomidine group demonstrated a notable increase in the T3 Mini-Cog© scores from the day of the surgery to T3, indicating a significant improvement in postoperative cognitive function. Conversely, the control group showed a decline in the aforementioned score. These results, together, indicate that the pre- and postoperative Mini-Cog© scores can help screen cognitive impairment in elderly patients previously undiagnosed with dementia or at greater risk of developing PND. The application of the Mini-Cog© test enhanced its utility in settings where a patient manifests delirium-like symptoms upon emerging from anesthesia. The nurse-to-patient ratio increases when these patients are transferred from the recovery room to the ward, making it difficult to provide one-to-one treatment. Consequently, the early diagnosis of cognitive impairment in the ward becomes challenging, thereby increasing the likelihood of developing POCD. Furthermore, it is difficult to diagnose PND as neurocognitive symptoms fluctuate and are different from those experienced by patients immediately after the surgery. Therefore, the early detection of PND via the utilization of the Mini-Cog© test could be a crucial and cost-effective approach for enhancing patient outcomes and reducing the workload of nurses. In this respect, although no discernible disparity in the impact of dexmedetomidine on the HMGB1 levels was observed between the groups, the Mini-Cog© test is significant in that it provided comprehensive information to patients and their caregivers, in addition to the meticulous evaluation of their susceptibility to cognitive impairment during the perioperative period. This aspect of our research can be regarded as noteworthy.

Our study is the first to provide insights into the effects of dexmedetomidine on the HMGB1 levels and PND in humans. Previous studies only determined the associations between dexmedetomidine and PND or HMGB1 and PND [12,21,24,25]. The upregulation of HMGB1 levels within 24 h postoperatively indicates that the serum HMGB1 levels were the highest on postoperative day 1, as reported in other studies [35,36]. Preclinical studies in animal models reported that dexmedetomidine inhibits surgery-induced cognitive dysfunction and early inflammatory response initiated by HMGB1 [29,30,35,36]. Furthermore, the European Society of Anesthesiology recommends the use of dexmedetomidine to lower the risk of postoperative delirium [16]. However, clinical studies have demonstrated that dexmedetomidine did not reduce PND postoperatively despite the elevation in the HMGB1 levels on postoperative day 1 [28,37], which is consistent with the findings of our study. Nevertheless, the effect of dexmedetomidine in mitigating PND remains an ongoing issue requiring further study.

There may be several factors contributing to this result. First, the anti-inflammatory effect of dexmedetomidine may reduce the endotoxin-induced inflammatory response and inhibit proinflammatory cytokines, such as tumor necrosis factor (TNF)-α and interleukin (IL)-6 induced by surgical stress, which resulted in no difference in the HMGB1 levels between the groups. Healthy individuals have low HMGB1 levels, with a median of 1.3 ng/mL and a 95th percentile of 4.1 ng/mL [38]. The preoperative values obtained in our study corresponded well with the median reference range. An increase in the ∆(T1 − T2) HMGB1 levels and a decrease in the ∆(T2 − T3) HMGB1 levels was significantly greater in Group D, with a steep falling slope that recovered close to the baseline (Figure 4). However, the overall changes in the HMGB1 levels did not differ between the groups, suggesting that the anti-inflammatory effects of dexmedetomidine may have contributed to this result. Bulow et al. and Wu et al. reported a reduction in the increase in TNF-α and IL-6 levels during intraoperative dexmedetomidine infusion [39,40]. A previous meta-analysis demonstrated that intravenous anesthetics and analgesics can reduce the magnitude of the postoperative systemic inflammatory response [41]. In this study, the dexmedetomidine group demonstrated anesthetic and analgesic-sparing effects, including postoperative IV-PCA fentanyl dose similar to that reported in previous studies [42,43,44]. Thus, our results can be attributed to the anti-inflammatory effects of the attenuation of the surgical stress response via the administration of anesthetics and analgesics [41,45]. However, the result of this study showed opposite results, suggesting that other factors were involved. 

Second, the infusion rate and duration of administration have been established as important determinants [37]. Duan et al. reported that the optimal dose is a maintenance dose above 0.2 μg/kg/h with a loading dose of up to 0.5 μg/kg [46]. Prolonged administration may lead to increased undesirable effects of dexmedetomidine, such as hypotension and bradycardia. As the patients in this study were elderly and susceptible to these risks, the maintenance dose was reduced to 0.2 μg/kg/h. Thus, the dose was relatively less than the required dose compared with that in previous preclinical studies, leading to different results. 

Third, age- and sex-mediated differences may have contributed to the effects of HMGB1. The median age of our study population was 74 years old, which may have accounted for a particularly higher age. The oldest participant was aged 95 years, and an age of 80 years or older accounted for one-fifth of the study population, corresponding to 24 patients. Differences in the cellular neuroinflammatory response may also have contributed to age-dependent differences in BBB damage after TBI, that is, a more extensive breakdown of the BBB occurs in the pediatric brain after a pro-inflammatory insult [47]. The use of blood samples, not cellular samples, for investigating the release of HMGB1 and the high proportion of elderly participants in this study may have led to a conspicuous elevation in the HMGB1 levels not being observed. Zemskova et al. reported that the activation of necrotic cell death induced the release of HMGB1, resulting in a significant increase in HGMB1 levels in male but not female patients [48]. The results provide strong evidence of more activated inflammatory pathways in men that could predispose them to accelerated disease progression and poor survival prognosis. The proportion of female patients was higher, with a ratio of approximately 3:1 observed in this study; thus, the inter-group changes in the HMGB1 levels may not have been substantial. Consequently, it can be assumed that the release of inflammatory cytokines is affected by age and sex.

Thus, multiple factors are needed to reveal the effect of intraoperative dexmedetomidine infusion on cognitive function. The duration of the surgery, the overall physical condition of the patient, and patient characteristics (such as age, sex, and underlying disease, including ASA-PS), which can be confirmed via the results of multivariate analysis (Table 4), should be considered. We tried to separate the memory (three-item recall: 0–3) and visuoconstruction (clock drawing test: 0–2) functions of the T1 Mini-Cog test. The CDT in Mini-Cog relies on patient performance function related to vision, hearing, and motor skills. While there was no significant difference in the T1 Mini-Cog scores between the two groups, the scores of Group C were lower at all times. Given these results together, the higher presence of patients with poor vision or hearing and reduced motor skills in group C is one of the reasons. Therefore, we conducted separate statistical analyses of the Mini-Cog score according to each function area in both groups and did not find a significant difference between the groups (*p* = 0.211 for three-item recall and *p* = 0.276 for CDT). Data was not shown. Furthermore, the absolute mass (μg/kg) of dexmedetomidine, including the time and rate of injection that will not harm the patient, should also be considered. These aspects should be taken into account and thoroughly evaluated to obtain a comprehensive understanding of the effect of dexmedetomidine on the serum HMGB1 levels and incidence of POCD.

Our study has several limitations. First, our study was conducted at a single center, and patients were limited to those undergoing elective orthopedic surgery under general anesthesia. Therefore, the results may not be generalizable to all geriatric patients undergoing major surgery under general anesthesia. However, we aimed to reduce bias by selecting patients who underwent homogenous surgery. Second, since our study calculated the sample size based on the change in HMGB1 levels, it may not have been sufficient to show a difference in the incidence of delirium in our study due to the small sample size. Therefore, further studies must be conducted in the future with a large-scale randomized controlled trial.

While there was no significant difference observed in the HMGB1 levels between the two groups, this study holds significance due to the notable difference in the Mini-cog scores found in the dexmedetomidine group of elderly patients undergoing orthopedic surgery.

## 5. Conclusions

In conclusion, the intraoperative dexmedetomidine increased the postoperative Mini-Cog© score in elderly patients undergoing orthopedic surgery. Moreover, the Mini-Cog© score could aid in the early diagnosis, prevention, and treatment of PND during the perioperative period, suggesting that Mini-Cog©, which is easy, comfortable, and less time consuming for anesthesiologists and patients, can be a beneficial tool for evaluating the perioperative cognitive function.

## Figures and Tables

**Table 1:** Baseline patient characteristics in both groups.

Variables	Group C (*n* = 64)	Group D (*n* = 64)	*p*-Value
Sex (M/F)	17 (26.6%)/ 47 (73.4%)	20 (31.3%)/ 44 (68.8%)	0.559
Age (years)	74.23 ± 6.24	74.41 ± 6.00	0.874
BMI (kg/m^2^)	25.33 ± 3.81	25.74 ± 3.23	0.512
ASA-PS (1/2/3)	5/43/16	5/50/9	0.288
Educational level (elementary/middle/high/higher than college)	34/10/11/9	31/16/8/9	0.773
Length of hospital day	16.98 ± 11.16	15.61 ± 7.08	0.407
Diabetes mellitus	18 (28.1%)	15 (23.4%)	0.554
Hypertension	49 (76.6%)	53 (82.8%)	0.380
Cerebrovascular disease	3 (4.7%)	3 (4.7%)	0.670

Values are presented as mean ± SD or number (%). Student *t*-test is performed for continuous variables and chi-square test for categorical variables. The demographic characteristics do not significantly differ between the groups. BMI, body mass index; ASA-PS, American Society of Anesthesiologists physical status; Group C, control group; Group D, dexmedetomidine group.

**Table 2:** Perioperative variables.

Variables	Group C (*n* = 64)	Group D (*n* = 64)	*p*-Value
Total dose of the study drug (mL) *	23.25 ± 7.64	22.06 ± 5.84	0.322
Total dose of propofol (mg)	74.38 ± 23.36	59.84 ± 16.67	<0.001
Total dose of fentanyl (μg)	141.51 ± 57.60	109.45 ± 32.94	<0.001
Use of vasopressor (n)	27 (42.2%)	44 (68.8%)	0.002
Operation time (min)	116.33 ± 60.33	127.58 ± 58.80	0.287
Anesthesia time (min)	165.39 ± 64.17	180.86 ± 61.65	0.167
Total administered fluid (L)	1.45 ± 1.01	1.60 ± 0.92	0.385
Estimated blood loss (mL)	388.20 ± 363.75	408.59 ± 329.42	0.740
Delirium (n)	10 (15.63%)	4 (6.25%)	0.089
Postoperative day 1 NRS	2.81 ± 1.89	3.00 ± 1.85	0.574
PONV (n)	9 (14.1%)	8 (12.9%)	0.849
Total infused PCA dose (μg) ^†^	338.99 ± 104.04	277.56 ± 133.03	0.005

Values are presented as mean ± SD or number (%). Student’s *t*-test is performed for continuous variables; the chi-square test is performed for categorical variables. NRS, numeric rating scale; PCA, patient-controlled analgesia; PONV, postoperative nausea and vomiting; Group C, control group; Group D, dexmedetomidine group. * Saline for Group C and dexmedetomidine for Group D. ^†^ Established with a total of 500 μg fentanyl in 60 mL, which is 8.33 μg/mL fentanyl, programmed to deliver a bolus of 0.5 mL with 15 min lockout intervals and a background infusion of 0.5 mL/h.

**Table 3:** The Mini-Cog© score and Serum HMGB1 levels during the perioperative period.

	Group C (*n* = 64)	Group D (*n* = 64)	*p*-Value
Mini-Cog© score			
T1	3.72 ± 1.19	4.03 ± 1.13	0.129
T2	3.69 ± 1.14	4.08 ± 1.15	0.055
T3	3.44 ± 1.40	4.20 ± 1.06	0.001
Serum HMGB1 levels			
T1	2.22 ± 3.96	2.21 ± 3.87	0.984
T2	8.71 ± 5.94	9.86 ± 5.87	0.331
T3	7.97 ± 6.40	6.90 ± 5.86	0.382
Absolute difference in the serum HMGB1 levels			
$∆\left(T1-T2\right)$	6.488 ± 6.26	7.658 ± 6.26	<0.001
$∆\left(T1-T3\right)$	5.745 ± 6.40	4.696 ± 6.40	<0.001
∆(T2−T3)	0.743 ± 7.14	2.963 ± 7.14	0.004

The values are presented as mean ± SD. Student’s *t*-test is performed for the Mini-Cog© score and serum HMGB1 levels at T1, T2, T3, and absolute differences. The Mini-Cog© score of Group D at T3 is significantly higher than that of Group C. $∆\left(T2-T3\right)$ of HMGB1 level shows that the decline slope in the Group D is steeper than in the Group C significantly. HMGB1, high-mobility group box 1; T1, before the surgery; T2, postoperative day 1; T3, postoperative day 3; Group C, control group; Group D, dexmedetomidine group.

**Table 4:** Logistic regression for association of PND.

Variables	Univariate Analysis			Multivariate Analysis		
	Odds Ratio	95% CI	*p*-Value	Odds Ratio	95% CI	*p*-Value
Educational level	1.01	0.62–1.67	0.959	1.21	0.71–2.07	0.494
ASA-PS	3.76	1.26–11.20	0.017	4.68	1.29–17.01	0.019
Diabetes mellitus	3.39	1.09–10.53	0.035	2.53	0.65–9.82	0.181
Anesthesia time	1.00	0.99–1.01	0.423	1.00	0.99–1.01	0.817
T2 Mini-Cog© score	0.60	0.38–0.94	0.027	1.16	0.56–2.41	0.688
T3 Mini-Cog© score	0.52	0.35–0.77	0.001	0.48	0.26–0.88	0.017
T3 HMGB1 level	1.04	0.96–1.13	0.339	1.05	0.95–1.16	0.323
Infusion of dexmedetomidine	0.36	0.11–1.22	0.100	0.79	0.187–3.35	0.748

Multivariate logistic regression analysis is performed after adjusting for confounding factors using univariate logistic regression analysis. PND, perioperative neurocognitive disorders; CI, confidence interval; ASA-PS, American Society of Anesthesiologists physical status; HMGB1, high-mobility group box 1; T1, before surgery; T2, postoperative day 1; T3, postoperative day 3.

## Data Availability

The datasets generated during this current study are not publicly available but are available from the corresponding author upon reasonable request.

## Supplementary Materials

The following supporting information can be downloaded at: https://www.mdpi.com/article/10.3390/jcm12206610/s1, Supplementary S1: Mini-Cog instrument Form; Supplementary S2: CAM-ICU worksheet.
