# Supplementary material for: The Effect of Dexmedetomidine on the Mini-Cog Score and High-Mobility Group Box 1 Levels in Elderly Patients with Postoperative Neurocognitive Disorders Undergoing Orthopedic Surgery
Source: J Clin Med. 2023 Oct 19;12(20):6610. doi: 10.3390/jcm12206610 (PMC10607676; doi:10.3390/jcm12206610)

Supplementary S1. Mini-Cog instrument Form

Mini-Cog©

Instructions for Administration & Scoring

ID:__________ Date: ______________

Step 1: Three Word Registration

Look directly at person and say, "Please listen carefully. I am going to say three words that I want you to repeat back to me now and try to remember. The words are [select a list of words from the versions below]. Please say them for me now." If the person is unable to repeat the words after three attempts, move on to Step 2 (clock drawing).

The following and other word lists have been used in one or more clinical studies.[1-3] For repeated administrations, use of an alternative word list is recommended.

Version 1	Version 2	Version 3	Version 4	Version 5	Version 6
Banana	Leader	Village	River	Captain	Daughter
Sunrise	Season	Kitchen	Nation	Garden	Heaven
Chair	Table	Baby	Finger	Picture	Mountain

Step 2: Clock Drawing

Say: "Next, I want you to draw a clock for me. First, put in all of the numbers where they go." When that is completed, say: "Now, set the hands to 10 past 11."

Use preprinted circle (see next page) for this exercise. Repeat instructions as needed as this is not a memory test. Move to Step 3 if the clock is not complete within three minutes.

Step 3: Three Word Recall

Ask the person to recall the three words you stated in Step 1. Say: "What were the three words I asked you to remember?" Record the word list version number and the person's answers below.

Word List Version:________ Person's Answers: ______________ ______________ ______________

Scoring

Word Recall: ______ (0-3 points)	1 point for each word spontaneously recalled without cueing.
Clock Draw: ______ (0 or 2 points)	Normal clock = 2 points. A normal clock has all numbers placed in the correct sequence and approximately correct position (e.g., 12, 3, 6 and 9 are in anchor positions) with no missing or duplicate numbers. Hands are pointing to the 11 and 2 (11:10). Hand length is not scored.<br>Inability or refusal to draw a clock (abnormal) = 0 points.
Total Score: ______ (0-5 points)	Total score = Word Recall score + Clock Draw score.<br><br>A cut point of <3 on the Mini-Cog™ has been validated for dementia screening, but many individuals with clinically meaningful cognitive impairment will score higher. When greater sensitivity is desired, a cut point of <4 is recommended as it may indicate a need for further evaluation of cognitive status.

Clock Drawing

ID:____________ Date:_________________

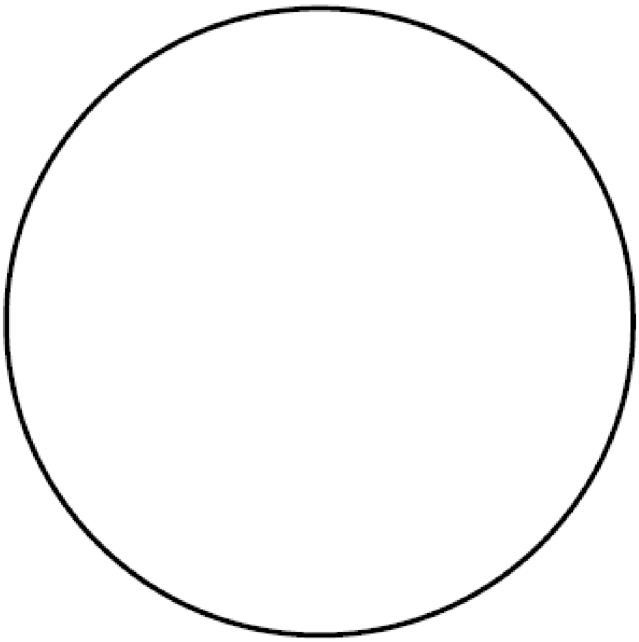

Supplement: Supplementary file 1 [file jcm-12-06610-s001.zip › Supplementary S1.pdf]
