# Supplementary material for: The Effect of Dexmedetomidine on the Mini-Cog Score and High-Mobility Group Box 1 Levels in Elderly Patients with Postoperative Neurocognitive Disorders Undergoing Orthopedic Surgery
Source: J Clin Med. 2023 Oct 19;12(20):6610. doi: 10.3390/jcm12206610 (PMC10607676; doi:10.3390/jcm12206610)
Supplement: Supplementary file 1 [file jcm-12-06610-s001.zip › Supplementary S2.pdf]

Supplementary S2. CAM-ICU worksheet

CAM-ICU Worksheet

Feature 1: Acute Onset or Fluctuating Course	Score	Check here if Present
Is the pt different than his/her baseline mental status?<br>OR<br>Has the patient had any fluctuation in mental status in the past 24 hours as evidenced by fluctuation on a sedation scale (i.e., RASS), GCS, or previous delirium assessment?	Either question Yes →	☐
**Feature 2: Inattention**		
Letters Attention Test (See training manual for alternate Pictures)<br><br>Directions: Say to the patient, *"I am going to read you a series of 10 letters. Whenever you hear the letter 'A,' indicate by squeezing my hand."* Read letters from the following letter list in a normal tone 3 seconds apart.<br><br>**S A V E A H A A R T**<br><br>**Errors are counted when patient fails to squeeze on the letter "A" and when the patient squeezes on any letter other than "A."**	Number of Errors >2 →	☐
**Feature 3: Altered Level of Consciousness**		
Present if the Actual RASS score is anything other than alert and calm (zero)	RASS anything other than zero →	☐
**Feature 4:Disorganized Thinking**		
Yes/No Questions (See training manual for alternate set of questions)<br><br>1. Will a stone float on water?<br>2. Are there fish in the sea?<br>3. Does one pound weigh more than two pounds?<br>4. Can you use a hammer to pound a nail?<br><br>**Errors are counted when the patient incorrectly answers a question.**<br><br>Command<br>Say to patient: "Hold up this many fingers" (Hold 2 fingers in front of patient) "Now do the same thing with the other hand" (Do not repeat number of fingers) *If pt is unable to move both arms, for 2nd part of command ask patient to "Add one more finger"<br><br>**An error is counted if patient is unable to complete the entire command.**	Combined number of errors >1→	☐

**Overall CAM-ICU**<br><br>**Feature 1 plus 2 and either 3 or 4 present = CAM-ICU positive**	**Criteria Met →**	☐ **CAM-ICU Positive** (Delirium Present)
	**Criteria Not Met →**	☐ **CAM-ICU Negative** (No Delirium)

Copyright © 2002, E. Wesley Ely, MD, MPH and Vanderbilt University, all rights reserved
